# Supplementary material for: The Effect of Vitamin K2 Supplementation on PIVKA-II Levels in Patients with Severe Motor and Intellectual Disabilities Undergoing Long-Term Tube Feeding
Source: Nutrients. 2023 Oct 25;15(21):4525. doi: 10.3390/nu15214525 (PMC10647752; doi:10.3390/nu15214525)
Supplement: Supplementary file 1 [file nutrients-15-04525-s001.zip › nutrients-2602365-supplementary.pdf]

Table S1. List of abbreviations.

Abbreviations	Contents
SMID	Severe motor and intellectual disabilities
PIVKA-II	Protein-induced vitamin K absence or antagonist II (prothrombin)
ucOC	Undercarboxylated osteocalcin
EM	Erythromycin
CAM	Clarithromycin
ST	Trimethoprim–sulfamethoxazole
PT	Prothrombin time
PT-INR	Prothrombin international ratio
aPTT	Activated partial thromboplastin time
AT-III	Antithrombin III
HDL	High-density lipoprotein
LDL	Low-density lipoprotein

Table S2. List of enteral formulas.

Macro- and Micro-nutrients	Enteral Formulas					Trace Minerals	
	Elental P® (/100 kcaL)	Isocal 1.0 Junior® (/100 kcaL)	Climeal® (/100 kcaL)	Meiflow® (/100 kcaL)	MeiBalance® (/100 kcaL)	TESON® (20 kcaL/125 mL)	V CRESC CP10® (80 kcaL/125 mL)
Vitamin K (μg)	4.49	9.0	8.0	6.3	5.0	0.0	0.0
Vitamin A (μg)	103.8	63	80	75	60	0.0	0.0
Vitamin D (μg)	2.7	1.0	1.2	0.63	0.50	0.0	0.0
Vitamin E (mg)	1.6	0.9	1.4	3.8	3.0	0.0	0.0
Protein (g)	3.3	2.8	3.75	4.0	5.0	0~13	12.0
Fat (g)	0.89	3.3	3.35	2.8	2.5	0.0	0.0
Carbohydrate (g)	19.9	14.4	14.65	15.9	15.5	4.5	4.5
Cupper (mg)	0.11	0.10	0.13	0.13	0.050	0.3	-
Zinc (mg)	0.9	3.0	6.0	6.0	6.0	20	50
Selenium (μg)	-	1.0	1.5	1.5	1.0	4.0	12.0
L-carnitine (mg)	-	20	-	-	20.0	150	-

Table S3. Recommended adequate intake of vitamin K and estimated energy requirement in Japan.

Population	Vitamin K (μg/day)		Estimated Energy Requirement (kcal/day)	
	Male	Female	Male	Female
0–5-month-old infants	4	4	950	900
6–11-month-old infants	7	7	1300	1250
1–2-year-old children	50	60	1550	1450
3–5-year-old children	60	70	1850	1700
6–7-year-old children	80	90	2250	2100
8–9-year-old children	90	110	2600	2400
10–11-year-old children	110	140	2800	2300
12–14-year-old children	140	170	2650	2000
15–17-year-old boys and girls	160	150	2700	2050

18–29-year-old men and women	150	150	2600	1950
30–49-year-old men and women	150	150	2400	1850
50–64-year-old men and women	150	150	2100	1650
65–74-year-old men and women	150	150	950	900
>75-year-old men and women	150	150	1300	1250

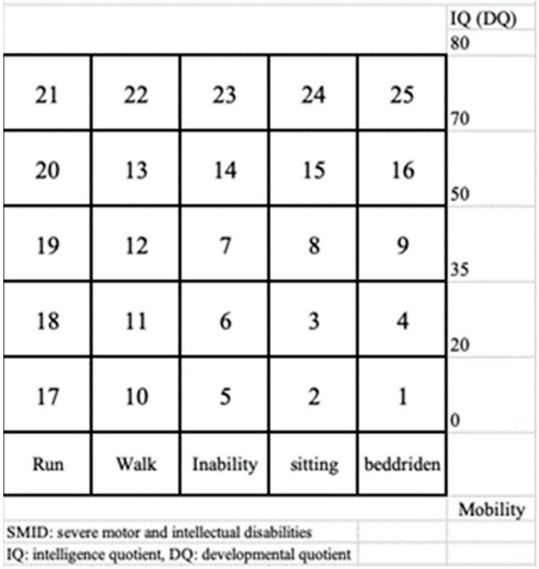

Figure S1. Ohshima’s Classification for SMID.
